# The Growth and Physiological Metabolic Responses of *Cunninghamia lanceolata* Saplings to Interactive Soil Acidification and Nitrogen Application and the Dominant Limiting Factor Determination

**DOI:** 10.3390/plants15213248

**Published:** 2026-10-22

**Authors:** Tingyu Sun, Jinchi Zhang, Qinyu Li, Yuhao Wang, Xin Liu

**Affiliations:** 1 College of Forestry Engineering, Shandong Agriculture and Engineering University, Jinan 250100, China; suntingyunjfu@163.com; 2 Co-Innovation Center for Sustainable Forestry in Southern China, Jiangsu Province Key Laboratory of Soil and Water Conservation and Ecological Restoration, Nanjing Forestry University, 159 Longpan Road, Nanjing 210037, China

**Keywords:** *Cunninghamia lanceolata*, soil acidification, nitrogen regulation, growth characteristics, physiological metabolism

## Abstract

To reveal the relative effects and interaction mechanism of soil acidification and nitrogen regulation on the growth of Chinese fir (*Cunninghamia lanceolata*) and to provide a scientific basis for the prevention and control of Chinese fir plantation decline, we conducted an 18-month greenhouse pot experiment using 1-year-old Chinese fir saplings. The experiment combined three acid solution spraying gradients to simulate soil acidification (control S0, distilled water; low acidification LS, acid solution pH 4.5; high acidification HS, acid solution pH 3.5; the initial soil pH was 6.3; acidification was simulated by spraying a mixed acid solution of H_2_SO_4_ and HNO_3_ at a molar ratio of 5:1) with three nitrogen application levels (no nitrogen N0, 0; low nitrogen LN, 50 mg N·kg^−1^ dry soil; high nitrogen HN, 200 mg N·kg^−1^ dry soil), forming nine treatment groups in total. Correlation analysis and a structural equation model (SEM) were used to analyze the influencing pathways. Soil acidification significantly reduced soil pH and nitrate nitrogen content, inhibited sapling growth, impaired the function of the photosynthetic system, and excessively activated superoxide dismutase (SOD) activity, which showed an extremely significant negative correlation with growth rate (−0.83). The total inhibitory effect of soil acidification on sapling growth reached 0.792, operating through direct growth inhibition, impaired nutrient supply, and enhanced antioxidant defense responses. Nitrogen application significantly increased soil available nitrogen content and alleviated stress under low acidification conditions by improving chlorophyll synthesis and photosynthetic efficiency; however, it had no significant direct effect on growth rate (0.15) and slightly exacerbated soil acidification (−0.45). The alleviating effect of nitrogen was completely lost under high acidification treatment with acid solution pH 3.5. The structural equation model showed a good fit (*p* = 0.379, GFI = 0.940, RMSEA = 0.051), confirming that soil acidification was the core dominant factor restricting Chinese fir growth. These results provide theoretical support and technical reference for precise nutrient management and decline control of Chinese fir plantations in southern China.

## 1. Introduction

Chinese fir (*Cunninghamia lanceolata*) belongs to the genus *Cunninghamia* of the family Taxodiaceae (Cupressaceae sensu lato). Although it is a unique fast-growing timber species endemic to the subtropical regions of southern China, it remains relatively unfamiliar to the international forestry community. Owing to the advantages of rapid growth, excellent wood quality, and wide adaptability, it has become the core tree species for plantation cultivation in this region. It plays an irreplaceable role in ensuring national timber security, enhancing forest carbon sink function, and maintaining regional ecological balance [1]. However, with the extension of plantation cultivation cycles and the widespread implementation of continuous planting systems, the decline of Chinese fir plantations has become increasingly prominent [2]. This decline is mainly manifested as reduced tree growth rate, decreased stand productivity, and frequent pest and disease outbreaks [1], which severely restricts the sustainable development of the Chinese fir plantation industry. Numerous studies have demonstrated that the decline of Chinese fir plantations is the result of a combination of multiple factors. Among them, the deterioration of soil physicochemical properties and the imbalance of nutrient supply are the core driving factors [3,4]. Soil acidification and insufficient available nitrogen supply are the most prominent issues [5], which jointly affect the growth, development, and physiological metabolism of Chinese fir [6].

Soil acidification is a widespread soil degradation problem in the process of plantation cultivation in the red soil region of southern China [7]. Long-term management of Chinese fir plantations accelerates the soil acidification process through litter decomposition, root exudate release, and atmospheric deposition [6,8,9]. The decrease in soil pH not only damages soil aggregate structure [10], but also significantly affects the availability of soil nutrients [11,12]. For example, it reduces the release efficiency of mineral nutrients such as available phosphorus and available potassium [13], and increases the activity of toxic metal ions such as aluminum and manganese [14]. These effects further inhibit the absorption and transport of nutrients by tree roots. Meanwhile, nitrogen is a core macronutrient required for plant growth and development. It directly participates in key physiological processes such as photosynthesis and protein synthesis [15,16]. Long-term continuous planting of Chinese fir plantations leads to excessive consumption of soil nitrogen and insufficient supply of available nitrogen (ammonium nitrogen and nitrate nitrogen) [5]. This further limits the height growth, leaf chlorophyll synthesis, and the increase in stress resistance-related enzyme activities of Chinese fir [17]. At present, numerous studies have reported the effects of soil acidification or insufficient available nitrogen supply on Chinese fir growth [12,18,19]. However, most of them focus on the effect of a single factor. In actual forest ecosystems, soil acidification and insufficient available nitrogen supply often coexist. The comprehensive mechanism of their interaction on the growth and development of Chinese fir is still unclear [3,20]. In addition, there is a lack of systematic quantitative comparison of the relative importance of the two factors to Chinese fir growth. This gap greatly restricts the research and development of precise regulation technologies for the decline of Chinese fir plantations. Mechanistically, soil acidification and nitrogen availability are closely coupled: soil acidification suppresses the activity of nitrifying microorganisms and alters the transformation of nitrogen forms, whereas nitrogen fertilization, through nitrification-induced proton release, can further acidify the soil; the net effect of this feedback on Chinese fir growth remains unclear.

Clarifying the relative effects and interaction mechanism of soil acidification and available nitrogen supply on Chinese fir growth is the prerequisite for formulating scientific and reasonable prevention and control measures for Chinese fir plantation decline. Based on previous findings, we proposed two testable hypotheses: (1) Soil acidification, by lowering soil pH, reducing soil nitrogen availability, and triggering physiological defense responses, exerts a stronger inhibitory effect on the growth of young Chinese fir than the promoting effect of nitrogen addition and is therefore the dominant limiting factor. (2) Nitrogen addition can alleviate the inhibitory effect of mild acidification on growth, but fails to mitigate growth suppression under severe acidification. To test these hypotheses, we conducted a soil acidification and nitrogen regulation experiment using Chinese fir saplings. The experiment included control, low acidification, high acidification, low nitrogen, high nitrogen, and their interactive combinations. We systematically determined the soil physicochemical indexes, as well as the growth, physiological and biochemical indexes of Chinese fir saplings. Through comprehensive analysis of multi-dimensional indexes, we revealed the independent and interactive effects of soil acidification and available nitrogen supply on the growth, development, and physiological metabolism of Chinese fir saplings. We also clarified the relative importance of the two factors affecting Chinese fir growth. This study provides a theoretical basis for in-depth analysis of the internal driving mechanism of Chinese fir plantation decline. Meanwhile, the results provide scientific and technical support for optimizing nutrient management strategies, regulating soil pH, improving stand productivity, and realizing sustainable management of Chinese fir plantations. It also has important practical significance for promoting the ecological construction and high-quality development of the forestry industry in southern China.

## 2. Materials and Methods

### 2.1. Plant Materials and Treatments

This study was carried out in a greenhouse at Xiashu Forest Farm of Nanjing Forestry University. We selected 1-year-old Chinese fir saplings with uniform height and ground diameter as the research materials. One-year-old saplings were chosen because this age class represents the sensitive early establishment stage of Chinese fir, when environmental stress has the most pronounced impact on survival and early growth performance; seedlings at this stage are also most commonly used in pot experiments to control initial conditions and reduce individual variation. It should be noted, however, that the stress responses of one-year-old seedlings may differ from those of older trees; therefore, caution is needed when extrapolating the present results to mature plantations.

The average height of the saplings was 31.3 cm, with an average ground diameter of 5.2 cm. In early March, the saplings were transplanted into plastic pots (40 cm height × 30 cm diameter). The cultivation substrate was yellow-brown soil collected from the forest area. The initial soil pH was 6.3, and the basic soil chemical properties were as follows: soil organic matter content 31.5 g·kg^−1^, available phosphorus 0.46 mg·kg^−1^, available potassium 58.28 mg·kg^−1^, ammonium nitrogen 3.05 mg·kg^−1^, and nitrate nitrogen 6.82 mg·kg^−1^. After transplanting, the saplings were placed in the greenhouse for a 2-month recovery period. During the recovery period, distilled water was irrigated weekly to maintain the soil field water capacity at 60–70%, ensuring the survival and environmental adaptation of the saplings.

After the recovery period, a completely randomized block design was adopted (pots were randomly positioned in the greenhouse and re-randomized monthly to minimize micro-environmental effects). We set three acid solution spraying levels to simulate soil acidification (control, S0; low acidification, LS; high acidification, HS) and three nitrogen addition levels (no nitrogen addition, N0; low nitrogen addition, LN; high nitrogen addition, HN), as well as their interactive treatments. A total of nine treatment groups were established, each with five replicates, giving 45 saplings in total. The pH gradients of acid solutions were set according to the range of soil pH observed in Chinese fir plantations of southern China, where long-term continuous planting has caused soil pH to decline from ~6.0 to 3.5–4.8 in severely degraded stands [3]. Acid solutions with pH 4.5 and 3.5 were sprayed to simulate mild and severe soil acidification scenarios, respectively. The nitrogen addition rates were set based on the background nitrogen deposition level (~30 kg N·ha^−1^·yr^−1^) in the subtropical region of China, with LN corresponding to ambient deposition and HN corresponding to high deposition scenarios.

The specific treatment design was as follows: ① soil-acidification-only treatment groups: no soil acidification (S0N0, sprayed with distilled water), low soil acidification (LSN0, sprayed with acid solution pH 4.5), high soil acidification (HSN0, sprayed with acid solution pH 3.5); ② low nitrogen treatment groups: low nitrogen addition (S0LN, sprayed with distilled water containing low-dose nitrogen fertilizer), LSLN (sprayed with acid solution pH 4.5 containing low-dose nitrogen fertilizer), HSLN (sprayed with acid solution pH 3.5 containing low-dose nitrogen fertilizer); and ③ high nitrogen treatment groups: S0HN (sprayed with distilled water containing high-dose nitrogen fertilizer), LSHN (sprayed with acid solution pH 4.5 containing high-dose nitrogen fertilizer), HSHN (sprayed with acid solution pH 3.5 containing high-dose nitrogen fertilizer). The complete treatment combinations are listed in Table S1 in the Supplementary Materials.

Soil acidification was simulated by spraying acid solutions. The acid solution was prepared by mixing 0.5 mol·L^−1^ H_2_SO_4_ and 0.5 mol·L^−1^ HNO_3_ at a molar ratio of 5:1, which mimics the typical ion composition of acid rain in southern China. We acknowledge that HNO_3_ introduces additional nitrogen along with protons; the nitrogen input from acid solutions was quantified and considered in the data interpretation. For the HS treatment, the annual nitrogen input via acid solution was approximately 2.1 g N·m^−2^, accounting for ~7% of the HN treatment nitrogen input, which was relatively small compared with the urea nitrogen gradient. The acid solution was diluted to pH 4.5 (LS) and 3.5 (HS) before spraying. The control group (S0) was sprayed with the same amount of distilled water (pH = 7.0).

Urea was selected as the nitrogen source because it is the most widely used nitrogen fertilizer in forestry production in China. We note that urea hydrolysis and subsequent nitrification can also induce soil acidification; this effect was considered an inherent property of nitrogen fertilization and was included in the interpretation of nitrogen treatment effects. The urea applied was of analytical reagent grade, with a nitrogen content of ≥46.4%. The nitrogen application rate was 50 mg N·kg^−1^ dry soil for low nitrogen (LN) treatment, and 200 mg N·kg^−1^ dry soil for high nitrogen (HN) treatment. Nitrogen fertilizer was applied in 3 splits at the initial, middle, and final stages of the treatment at a ratio of 1:1:1. It was dissolved in the spray solution and then irrigated into the soil. The spray frequency and total amount for all treatments were set with reference to the multi-year average annual precipitation of the study area. Spraying was performed 4 times a month, with a spray volume of 438.54 mL per sapling each time. The total spray volume during the experimental period was 670.38 mm. We note that the experimental acidification was applied at an accelerated rate compared with gradual natural acidification in the field; this allows testing of stress response mechanisms but may not fully replicate long-term field acclimation processes. During the treatment period, the greenhouse environment was controlled as follows: temperature 25 ± 3 °C, relative humidity 70% ± 5%, and natural light duration supplemented to 12 h·d^−1^. Weeds in the pots were removed regularly to avoid interference from pests and diseases. The greenhouse was equipped with automated temperature and humidity control and supplemental lighting systems, which maintained the set environmental conditions throughout the experimental period. At the end of the 18-month treatment, root growth was observed to fill most of the pot volume, indicating moderate root restriction; this effect was common across all treatments and was considered an inherent limitation of the pot experiment.

### 2.2. Determination of Soil Physicochemical Properties

After the experiment, topsoil samples (0–20 cm) were collected evenly from each pot in all treatment groups. After removing impurities such as roots, the soil samples were divided into two parts. One part of fresh soil was refrigerated at 4 °C for the determination of ammonium nitrogen and nitrate nitrogen contents. The other part was air-dried naturally and passed through a 2 mm sieve for the determination of soil pH, available phosphorus, and available potassium contents.

Soil pH (pH H_2_O) was determined by the potentiometric method in a soil–water suspension (soil:water ratio 1:2.5, *v*/*v*) with a pH meter. Available phosphorus was determined by the molybdenum–antimony anti-spectrophotometric method, and the absorbance was measured at a wavelength of 700 nm using an ultraviolet–visible spectrophotometer. Available potassium was determined by the flame photometric method with a flame photometer. Ammonium nitrogen and nitrate nitrogen were determined using a flow injection analyzer.

### 2.3. Determination of Plant Growth Parameters

The height of the saplings was measured at the initial stage (after transplanting recovery) and the final stage (18 months after treatment) of the experiment. The relative height growth rate (GR) was calculated using the formula: GR = (H_final_ − H_initial_)/H_initial_, where H_final_ is the final height (cm), and H_initial_ is the initial height after recovery (cm).

Chlorophyll content was determined following the method of Liu et al. (2018) [21]. At the end of the experiment, functional leaves (the 3rd to 5th leaves downward from the terminal bud) of saplings in each treatment group were selected. After removing the main vein, 0.1 g of fresh sample was weighed and placed in 10 mL of acetone–ethanol mixed extract (volume ratio 1:1). The sample was soaked in the dark for 10 h, with shaking once every 2 h. After extraction, the sample was centrifuged at 2500 r·min^−1^ for 20 min, and the supernatant was taken as the test solution. The absorbance was measured at wavelengths of 663 nm and 645 nm using an ultraviolet–visible scanning spectrophotometer (UV-VIS, UV-2700i Shimadzu Corporation, Kyoto, Japan).

Chlorophyll fluorescence parameters were determined using a chlorophyll fluorescence imaging system (CF Imager, Technologica Ltd., Colchester, UK) following the method of Liu et al. (2018) [21]. The same functional leaves used for chlorophyll determination were selected. Before measurement, the leaves were dark-adapted for 30 min. Then the minimum fluorescence (Fo) and maximum fluorescence (Fm) in the dark-adapted state were measured. After applying 500 μmol·m^−2^·s^−1^ actinic light for light adaptation, the steady-state fluorescence (Fs), light-adapted minimum fluorescence (Fo′), and light-adapted maximum fluorescence (Fm′) in the light-adapted state were measured. The maximum photochemical efficiency (Fv/Fm), actual photochemical efficiency (Fv′/Fm′), effective photochemical efficiency (ΦPSII), photochemical quenching coefficient (qP), and non-photochemical quenching coefficient (NPQ) were calculated according to the corresponding formulas.

Before the determination of antioxidant enzyme activities and malondialdehyde (MDA) content, the crude enzyme solution was prepared following the method of Liu et al. (2018) [21]. In total, 2–3 g of fresh functional leaves was selected, and 5 mL of ice-precooled phosphate buffer (50 mmol·L^−1^, pH = 7.8) was added. The sample was ground into homogenate in an ice bath, and then centrifuged at 15,000× *g* and 4 °C for 20 min. The supernatant was the crude enzyme extract, which was refrigerated at 4 °C for later use. Superoxide dismutase (SOD) activity was determined by the nitroblue tetrazolium (NBT) photoreduction method. One unit of enzyme activity was defined as the amount of enzyme required to inhibit 50% of NBT photoreduction. Peroxidase (POD) activity was determined by the guaiacol method. One unit of enzyme activity was defined as the amount of enzyme required to increase the absorbance by 0.01 per minute. Catalase (CAT) activity was determined by the ultraviolet absorption method. One unit of enzyme activity was defined as the amount of enzyme required to reduce the absorbance of H_2_O_2_ by 0.01 per minute. Malondialdehyde (MDA) content was determined by the thiobarbituric acid (TBA) method. The crude enzyme extract was mixed with TBA reagent, followed by a boiling water bath for 15 min. After cooling and centrifugation, the absorbance of the supernatant was measured at wavelengths of 532 nm and 600 nm. The MDA content was calculated according to its molar absorption coefficient.

### 2.4. Statistical Analysis

Statistical analyses were performed using SPSS 19.0 software. All data of measured indexes were expressed as mean ± standard error (mean ± SE, *n* = 5). The normality and homogeneity of variance of the data were tested first. After meeting the conditions, two-way analysis of variance (two-way ANOVA) was used to test the effects of nitrogen level (N), soil acidification gradient (S, represented by the pH of the sprayed acid solution), and their interaction (N × S) on soil physicochemical indexes, Chinese fir growth indexes, and physiological and biochemical indexes. If the main effect or interaction effect was significant (*p* < 0.05), Duncan’s multiple range test was used to analyze the significance of differences between groups. Pearson correlation analysis was performed using R 4.6.0 software to reveal the correlation between various factors and indexes. A structural equation model (SEM) was constructed using AMOS 24.0 software to explore the direct and indirect influencing pathways of nitrogen level (N) and soil acidification gradient (S, represented by the pH of the sprayed acid solution) on the growth rate of Chinese fir through soil physicochemical indexes and physiological and biochemical indexes. The model fit was evaluated by indicators such as the chi-square test (χ^2^) and root mean square error of approximation (RMSEA). All figures and tables were generated using Origin 9.0 software, and the significance level was set at *p* < 0.05.

## 3. Results

### 3.1. Responses of Soil Physicochemical Properties to Soil Acidification and Nitrogen Regulation

**Figure 1:**
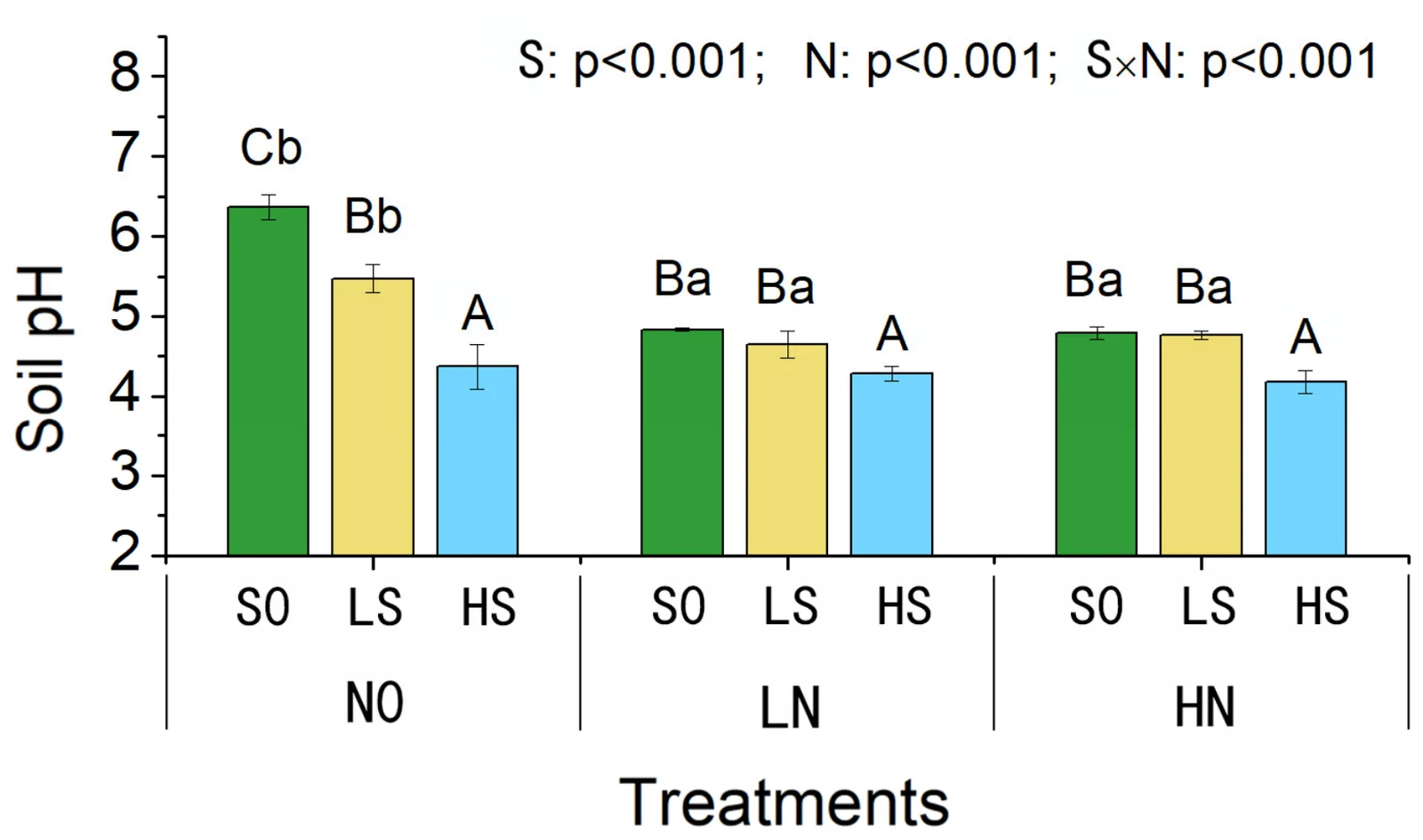
The effects of soil acidification (S) and nitrogen addition (N) on soil pH. The error bars are standard errors of the mean. The uppercase and lowercase letters above the bars indicate significant differences (*p* < 0.05) among the soil acidification levels and among the nitrogen addition levels, respectively; the bars without lowercase letters represent non-significant differences among the nitrogen addition levels (*p* > 0.05). S0, no soil acidification; LS, low soil acidification (acid solution pH 4.5); HS, high soil acidification (acid solution pH 3.5). N0, no nitrogen addition; LN, low nitrogen addition; HN, high nitrogen addition.

**Figure 2:**
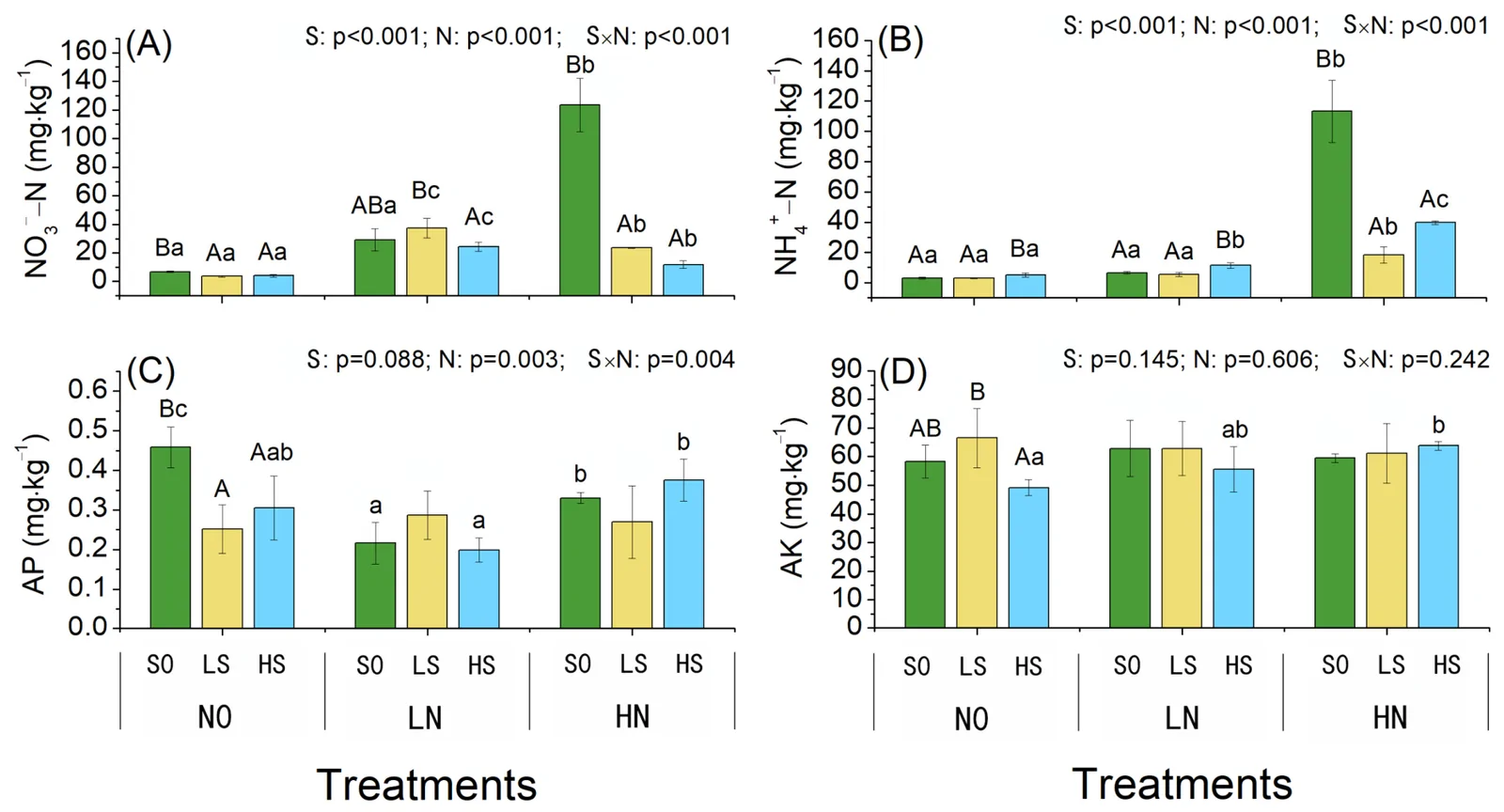
The effects of soil acidification (S) and nitrogen addition (N) on soil available nutrients. The error bars are standard errors of the mean. (**A**) NO_3_^−^-N, soil nitrate nitrogen; (**B**) NH_4_^+^-N, soil ammonium nitrogen; (**C**) AP, soil available phosphorus; (**D**) AK, soil available potassium.The uppercase and lowercase letters above the bars indicate significant differences (*p* < 0.05) among the soil acidification levels and among the nitrogen addition levels, respectively; the bars without letters represent non-significant differences between the treatments in the corresponding comparison group (*p* > 0.05). S0, no soil acidification; LS, low soil acidification; HS, high soil acidification. N0, no nitrogen addition; LN, low nitrogen addition; HN, high nitrogen addition.

The results of two-way ANOVA showed that soil acidification, nitrogen application, and their interaction had significant effects on soil pH, nitrate nitrogen (NO_3_^−^-N), and ammonium nitrogen (NH_4_^+^-N) contents (*p* < 0.05, Figure 1 and Figure 2A,B). The soil pH of the control treatment (S0N0) was significantly higher than those of low acidification (LS, sprayed with acid solution pH 4.5) and high acidification (HS, sprayed with acid solution pH 3.5) treatments (*p* < 0.05). The soil pH showed a significant downward trend with the increase in acidification degree (Figure 1). Under the low acidification level (acid solution pH 4.5), nitrogen application (LN, HN) significantly reduced soil pH (*p* < 0.05). Under the high acidification level (acid solution pH 3.5), nitrogen application had no significant effect on soil pH (*p* > 0.05).

Soil nitrate nitrogen content showed a downward trend with the increase in acidification degree at all nitrogen levels (Figure 2A). Ammonium nitrogen content increased significantly with the increase in acidification degree under no nitrogen (N0) and low nitrogen (LN) treatments (*p* < 0.05, Figure 2B). High nitrogen (HN) treatment significantly increased the basic contents of soil nitrate nitrogen and ammonium nitrogen (*p* < 0.05). However, under high nitrogen conditions, combined soil acidification treatment significantly reduced the contents of the two forms of available nitrogen (Figure 2A,B).

Nitrogen application had no significant effect on soil available potassium (AK) content under control (S0) and low acidification (LS, acid solution pH 4.5) conditions (*p* > 0.05, Figure 2D). Under high acidification (HS, acid solution pH 3.5) conditions, soil available potassium content increased significantly with the increase in nitrogen application level (*p* < 0.05). For soil available phosphorus (AP) content, acidification treatments (LS, HS) significantly reduced available phosphorus content under the no nitrogen (N0) condition (*p* < 0.05, Figure 2C). However, there was no significant difference in soil available phosphorus content among different acidification degrees under the low nitrogen (LN) and high nitrogen (HN) treatments (*p* > 0.05).

### 3.2. Responses of Chinese Fir Sapling Growth Rate to Soil Acidification and Nitrogen Regulation

**Figure 3:**
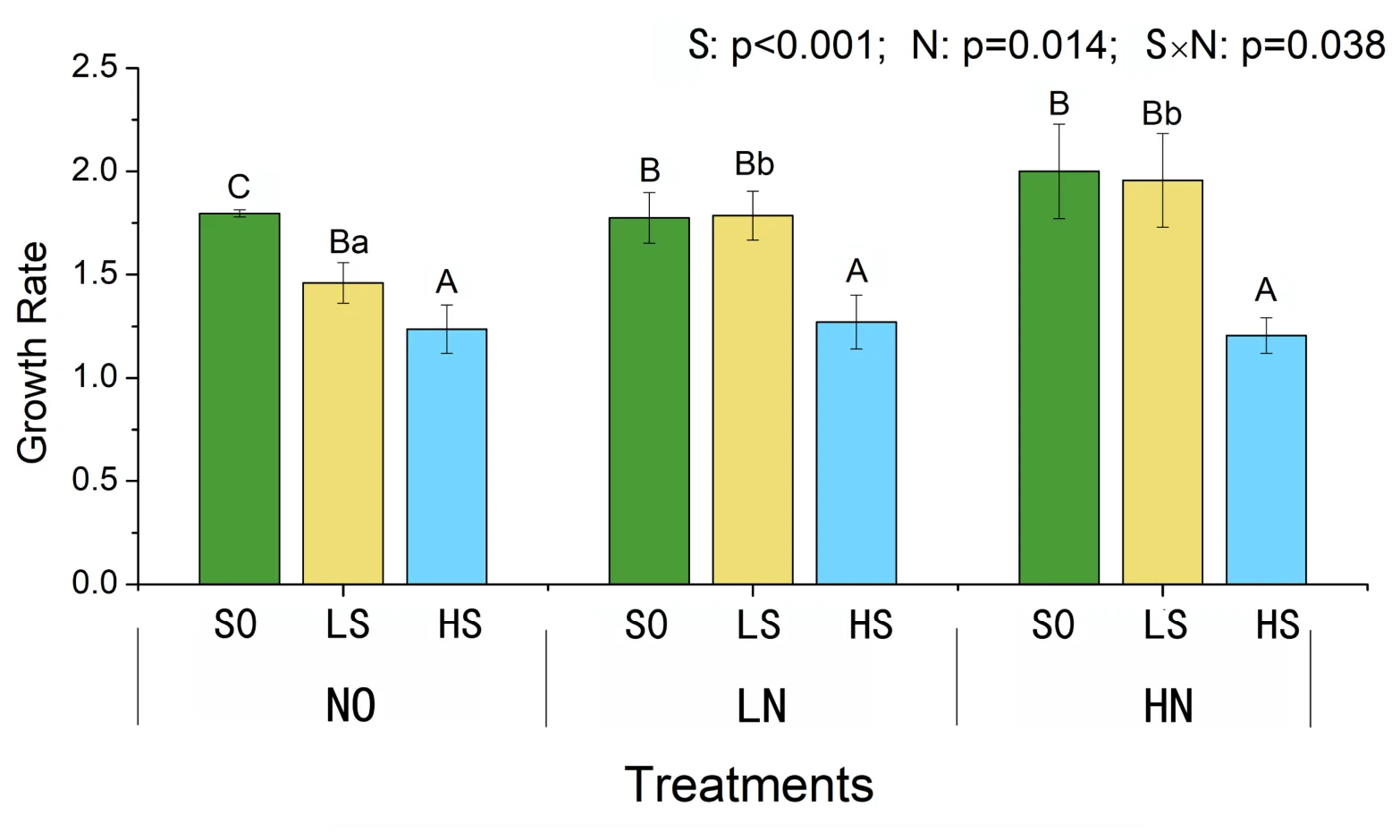
Changes in the tree growth rates under the different soil acidification (S) and nitrogen addition (N) treatments. The uppercase and lowercase letters above the bars indicate significant differences (*p* < 0.05) among the soil acidification levels and among the nitrogen addition levels, respectively; the bars without lowercase letters represent non-significant differences among the nitrogen addition levels (*p* > 0.05). S0, no soil acidification; LS, low soil acidification (acid solution pH 4.5); HS, high soil acidification (acid solution pH 3.5). N0, no nitrogen addition; LN, low nitrogen addition; HN, high nitrogen addition.

Soil acidification, nitrogen application, and their interaction had significant effects on the height growth rate (GR) of Chinese fir saplings (*p* < 0.05, Figure 3). Overall, the growth rate of Chinese fir saplings exhibited a significant decreasing trend with increasing soil acidification degree (*p* < 0.05). Under no acidification (S0) and low acidification (LS) conditions, the growth rate increased gradually with the increase of nitrogen application level. The growth rates of the LSLN and LSHN treatments were significantly higher than that of the single low acidification treatment (LSN0) (*p* < 0.05). However, under high acidification (HS) conditions, nitrogen application had no significant promoting effect on the growth rate of Chinese fir saplings (*p* > 0.05).

### 3.3. Responses of Chlorophyll Content and Chlorophyll Fluorescence Parameters to Soil Acidification and Nitrogen Regulation

**Figure 4:**
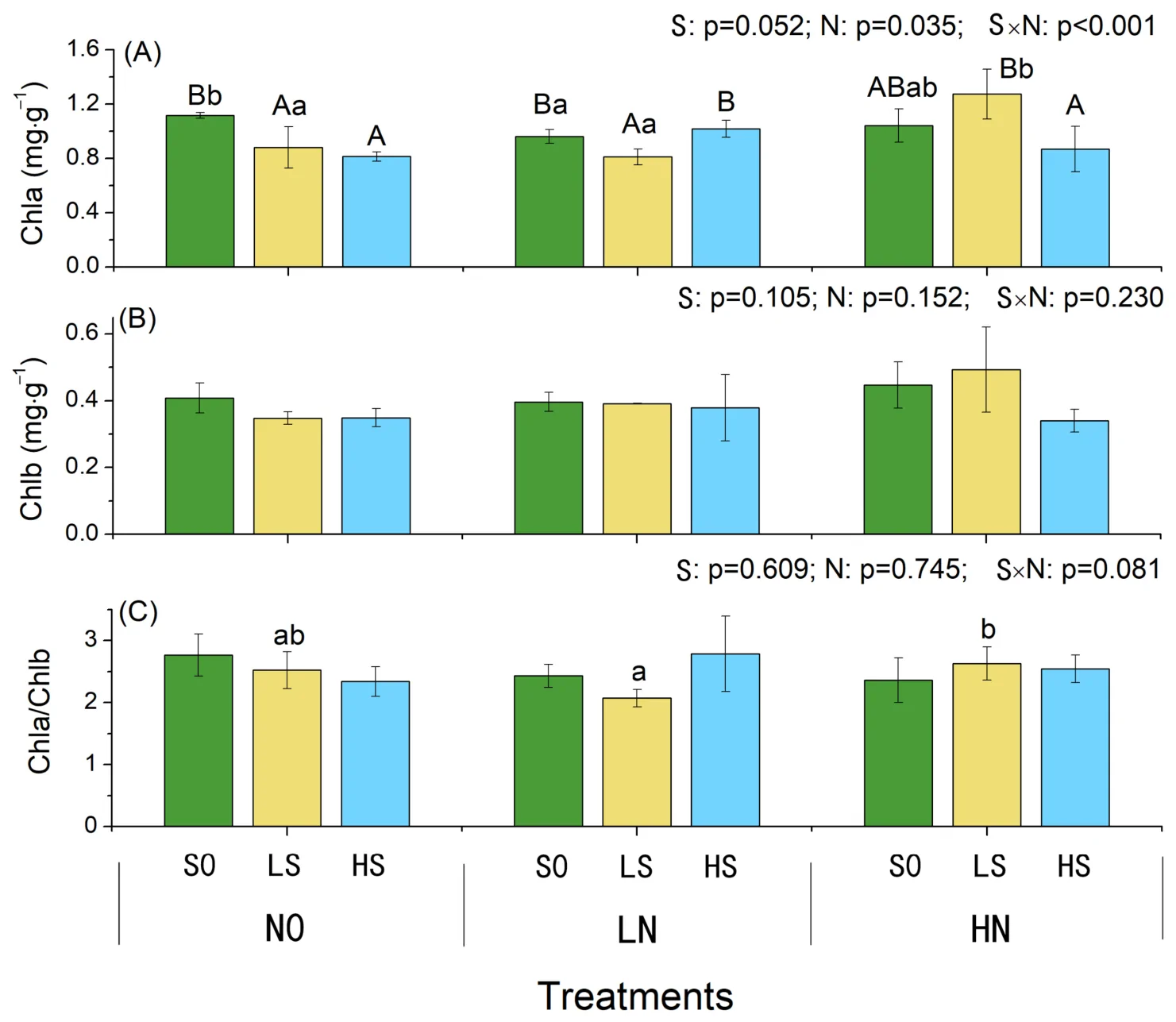
Changes in the leaf chlorophyll content under different soil acidification (S) and nitrogen addition (N) treatments. (**A**) chlorophyll a; (**B**) chlorophyll b; (**C**) the ratio of chlorophyll a to chlorophyll b. The uppercase and lowercase letters above the bars indicate significant differences (*p* < 0.05) among the soil acidification levels and among the nitrogen addition levels, respectively; the bars without letters represent non-significant differences between the treatments in the corresponding comparison group (*p* > 0.05). S0, no soil acidification; LS, low soil acidification (acid solution pH 4.5); HS, high soil acidification (acid solution pH 3.5). N0, no nitrogen addition; LN, low nitrogen addition; HN, high nitrogen addition.

Nitrogen application (*p* < 0.05) and its interaction with soil acidification (*p* < 0.001) had significant effects on chlorophyll a (Chla) content (Figure 4A). Under the no nitrogen treatment (N0), Chla content decreased significantly with the increase in acidification degree (*p* < 0.05). Under the low nitrogen treatment (LN), Chla content in the high acidification (HS) treatment was significantly higher than that in the low acidification (LS) treatment (*p* < 0.05). Under the high nitrogen treatment (HN), Chla content under the low acidification (LS) condition was significantly higher than that under the high acidification (HS) treatment (*p* < 0.05). Notably, soil acidification, nitrogen application, and their interaction had no significant effects on chlorophyll b (Chlb) content and chlorophyll a/b ratio (*p* > 0.05, Figure 4B,C).

Among the key chlorophyll fluorescence parameters, the response characteristics of PSII maximum photochemical efficiency (Fv/Fm), actual photochemical efficiency (Fv′/Fm′), effective photochemical efficiency (ΦPSII), photochemical quenching coefficient (qP), and non-photochemical quenching coefficient (NPQ) were different (Table S2). The high acidification (HS) treatment significantly reduced Fv/Fm and ΦPSII (*p* < 0.05), while nitrogen application alleviated this inhibitory effect. The alleviating effect of high nitrogen (HN) was better than that of low nitrogen (LN). qP showed a downward trend with the increase in acidification degree, and nitrogen application significantly increased the qP value under low acidification conditions (*p* < 0.05). NPQ showed an upward trend with the increase in acidification degree, and high nitrogen treatment significantly reduced the NPQ value under high acidification conditions (*p* < 0.05). Overall, the main effects of acidification and nitrogen addition on most fluorescence parameters were not statistically significant, whereas significant effects were mainly observed for their interaction; specifically, the high acidification treatment alone significantly reduced Fv/Fm and ΦPSII, and nitrogen application alleviated this inhibition. Among the measured parameters, the chlorophyll a response was most consistent with the growth pattern.

### 3.4. Responses of Antioxidant Enzyme Activities and Malondialdehyde (MDA) Content to Soil Acidification and Nitrogen Regulation

**Figure 5:**
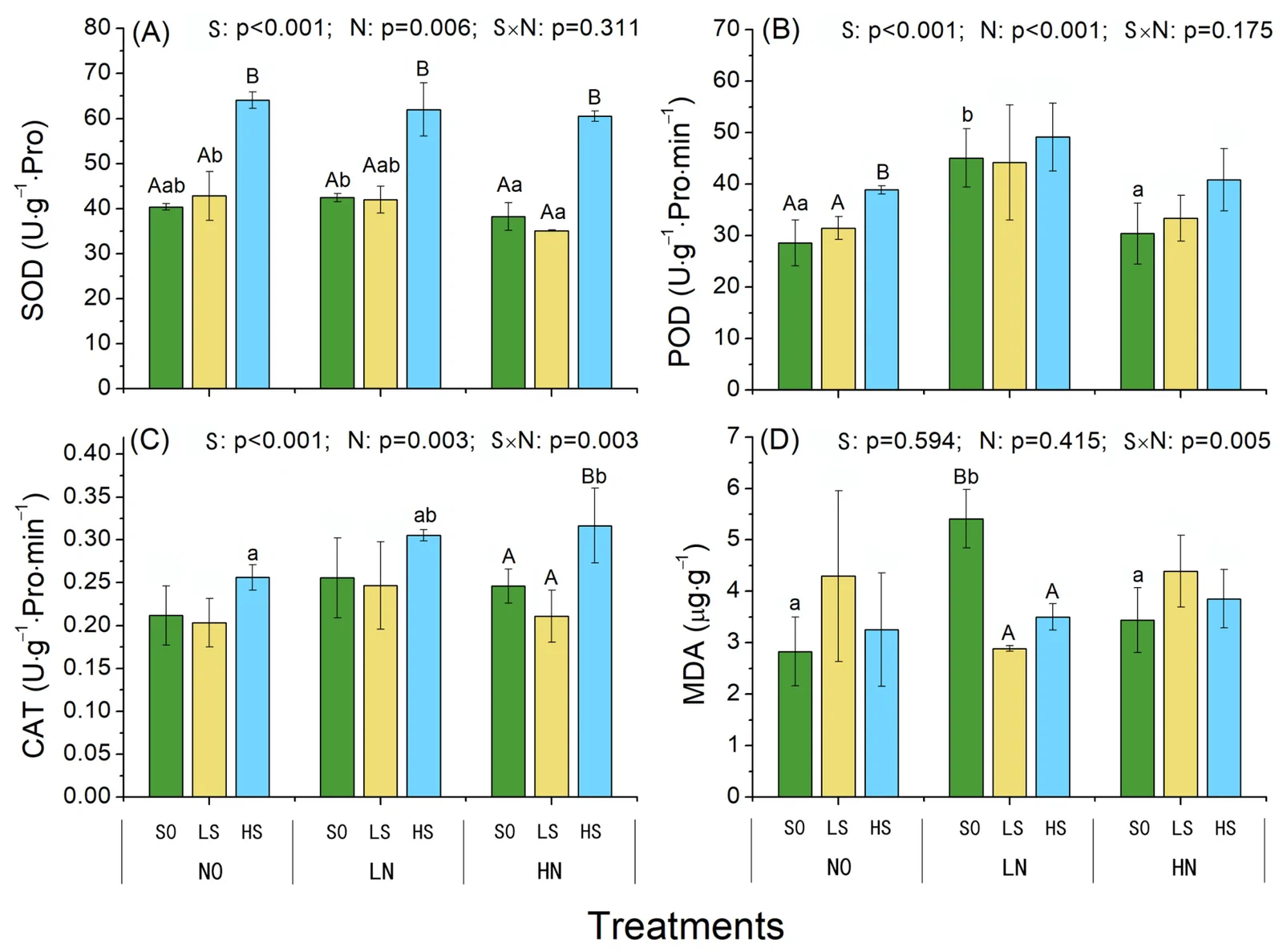
Changes in the activities of antioxidant enzymes under different soil acidification (S) and nitrogen addition (N) treatments. (**A**) SOD, superoxide dismutase; (**B**) POD, peroxidase; (**C**) CAT, catalase; (**D**) MDA, malondialdehyde. The uppercase and lowercase letters above the bars indicate significant differences (*p* < 0.05) among the soil acidification levels and among the nitrogen addition levels, respectively; the bars without letters represent non-significant differences between the treatments in the corresponding comparison group (*p* > 0.05). S0, no soil acidification; LS, low soil acidification (acid solution pH 4.5); HS, high soil acidification (acid solution pH 3.5). N0, no nitrogen addition; LN, low nitrogen addition; HN, high nitrogen addition; SOD, superoxide dismutase; POD, peroxidase; CAT, catalase; MDA, malondialdehyde.

The results of the two-way ANOVA showed that soil acidification and nitrogen application had significant effects on the activities of superoxide dismutase (SOD), peroxidase (POD), and catalase (CAT) in leaves (*p* < 0.05, Figure 5A–C). Their interaction only had significant effects on CAT activity and MDA content (*p* < 0.05, Figure 5C,D).

High acidification (HS) treatment significantly increased leaf SOD activity at all nitrogen levels (*p* < 0.05). Under the no acidification (S0) condition, SOD activity in the low nitrogen (LN) treatment was significantly higher than that in the high nitrogen (HN) treatment (*p* < 0.05). Under low acidification (LS) and high acidification (HS) conditions, SOD activity showed a gradual downward trend with the increase of nitrogen application level (Figure 5A).

Leaf POD activity increased gradually with the increase in soil acidification degree at all nitrogen levels. POD activity in the high acidification (HS) treatment was significantly higher than that in the low acidification (LS) and no acidification (S0) treatments (*p* < 0.05, Figure 5B). At all acidification levels, POD activity in the low nitrogen (LN) treatment was generally higher than that in the no nitrogen (N0) and high nitrogen (HN) treatments, but the significant difference was only observed under the no acidification (S0) condition (*p* < 0.05).

CAT activity showed a trend of first decreasing and then increasing with the increase in acidification degree, and reached the lowest value under the low acidification (LS) condition. The high nitrogen (HN) treatment significantly increased CAT activity under low and high acidification conditions (*p* < 0.05, Figure 5C). MDA content showed a trend of first increasing and then decreasing with the increase in acidification degree under the no nitrogen (N0) and high nitrogen (HN) treatments. MDA contents in the LSLN and HSLN treatments were significantly lower than that in the single low nitrogen treatment (S0LN) (*p* < 0.05). MDA content in the low nitrogen (LN) treatment was significantly higher than that in the no nitrogen (N0) and high nitrogen (HN) treatments (*p* < 0.05, Figure 5D). Overall, the increase in SOD and POD activities with increasing acidification indicates the activation of antioxidant defense responses; however, MDA content did not increase in parallel, suggesting that, under the present experimental conditions, acidification did not cause pronounced membrane lipid peroxidation damage, and the defense responses were not fully synchronized with the degree of oxidative damage.

### 3.5. Correlation Between Sapling Growth Rate and Chlorophyll Content, Chlorophyll Fluorescence, and Antioxidant Enzyme Activities

**Figure 6:**
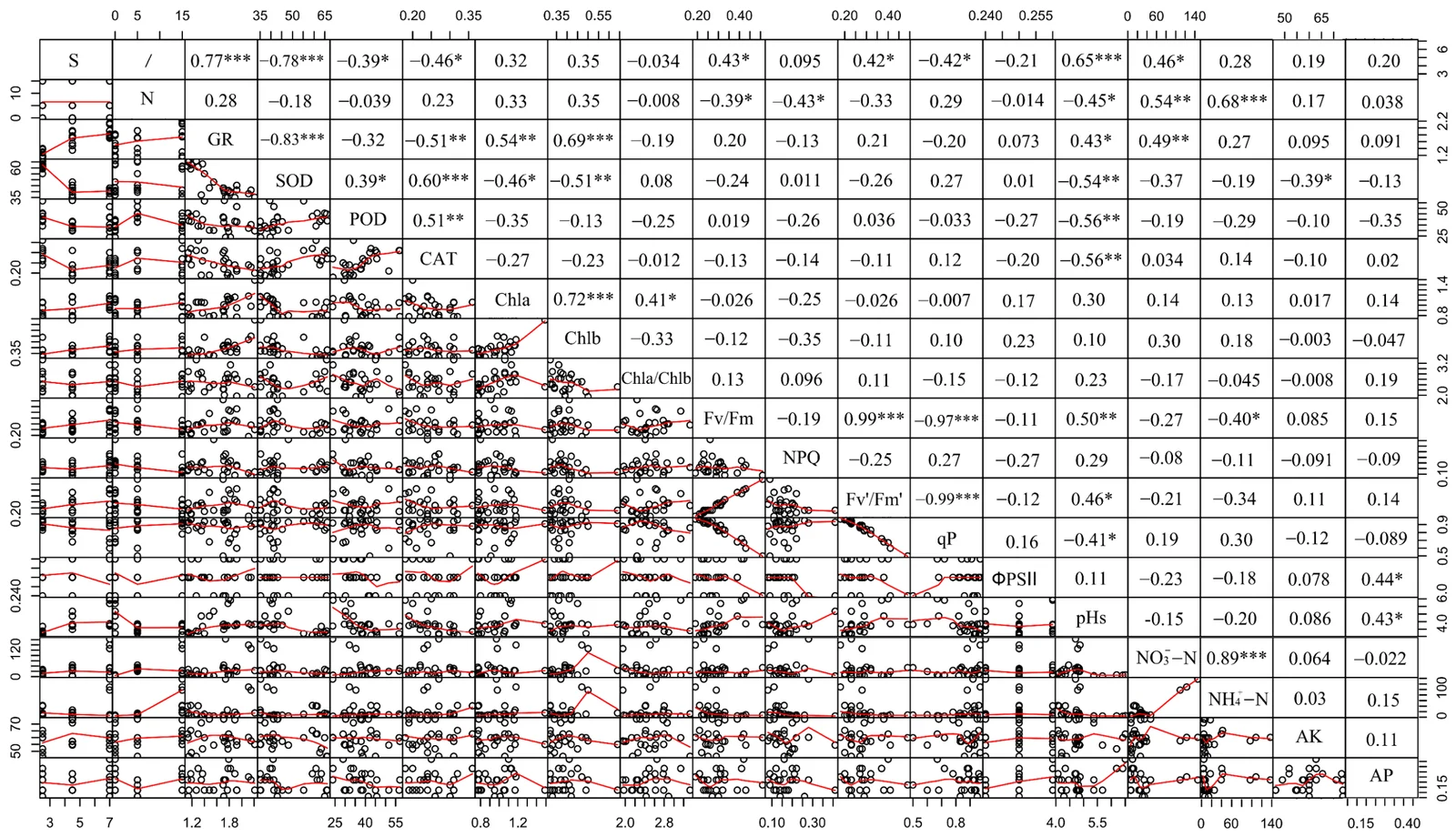
Correlation analysis. S, soil acidification gradient (represented by the pH of the sprayed acid solution; a higher value indicates lower soil acidification degree); N, nitrogen addition; GR, growth rate; SOD, superoxide dismutase; POD, peroxidase; CAT, catalase; Chla, chlorophyll a; Chlb, chlorophyll b; Chla/Chlb, ratio of chlorophyll a to chlorophyll b; Fv/Fm, maximum photochemical efficiency; NPQ, non-photochemical quenching coefficient; Fv′/Fm′, actual photochemical efficiency; qP, photochemical quenching coefficient; ΦPSII, effective photochemical efficiency; pH, soil pH value; NO_3_^−^-N, soil nitrate nitrogen; NH_4_^+^-N, ammonium nitrogen; AK, available potassium; AP, available phosphorus. The lower triangle displays pairwise scatter plots with red fitted trend lines to visualize bivariate relationship patterns, and the upper triangle presents the corresponding Pearson correlation coefficients, where *, **, and *** indicate statistical significance at *p* < 0.05, *p* < 0.01, and *p* < 0.001, respectively.

To visualize the overall relationships among soil acidification, nitrogen addition, sapling growth, and physiological indexes, Pearson correlation analysis was performed (Figure 6). The associations among the soil acidification gradient (S), nitrogen level (N), growth rate (GR), and antioxidant enzyme activities provide the basis for the subsequent path analysis. Correlation analysis (Figure 6) showed that the soil acidification gradient (S, represented by the pH of the sprayed acid solution) was extremely significantly positively correlated with soil pH (0.65 ***) and the growth rate (GR) of Chinese fir saplings (0.77 ***), and significantly negatively correlated with leaf SOD activity (−0.78 ***) and CAT activity (−0.46 *).That is, a lower soil acidification degree (higher pH of the sprayed acid solution) corresponded to higher soil pH, faster sapling growth, and lower antioxidant enzyme activities. Nitrogen addition level (N) was significantly positively correlated with soil nitrate nitrogen (0.54 **) and ammonium nitrogen (0.68 ***), but significantly negatively correlated with soil pH (−0.45 *), leaf fluorescence parameters Fv/Fm (−0.39 *) and NPQ (−0.43 *). Growth rate (GR) was significantly negatively correlated with leaf SOD activity (−0.83 ***) and CAT activity (−0.51 **), and significantly positively correlated with chlorophyll a and b contents (0.54 **, 0.69 ***), soil pH (0.43 *), and nitrate nitrogen content (0.49 **).

**Figure 7:**
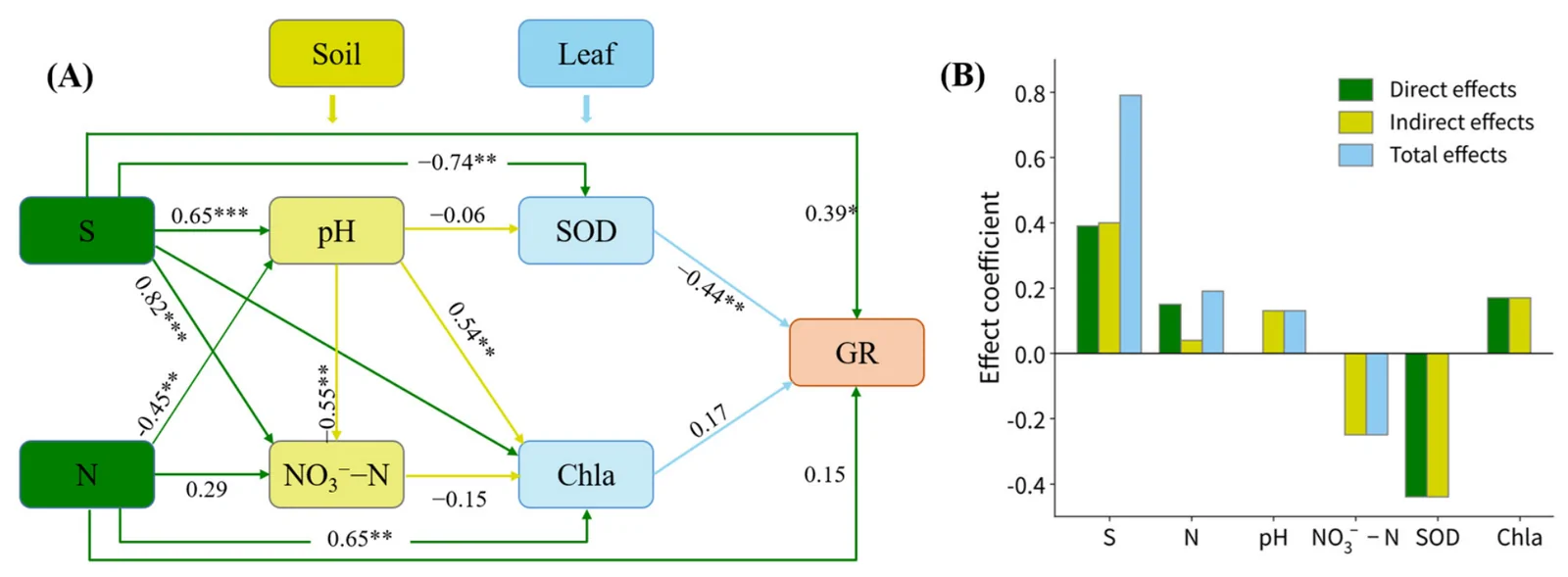
Structural equation models of the soil acidification gradient (S, represented by the pH of the sprayed acid solution) and nitrogen addition (N) effects on growth rate (GR) (*p* = 0.379 > 0.05; GFI = 0.940 > 0.900; RMSEA = 0.051). (**A**) Standardized path coefficients; (**B**) Direct, indirect, and total effects of each predictor on GR. The numbers on the arrows are standardized path coefficients; a higher S value represents lower soil acidification degree. pH, soil pH value; NO_3_^−^-N, soil nitrate nitrogen; SOD, superoxide dismutase; Chla, chlorophyll a; *, *p* < 0.05; **, *p* < 0.01; ***, *p* < 0.001.

The constructed structural equation model had a good fit (*p* = 0.379; GFI = 0.940; RMSEA = 0.051), which could effectively reveal the influencing pathways (Figure 7). The soil acidification gradient (S) had a significant direct positive effect on growth rate (GR), and also exerted an indirect promoting effect by increasing soil pH and reducing SOD activity, with a total effect value of 0.792. From the perspective of soil acidification stress, this means that acidification had strong direct and indirect inhibitory effects on sapling growth, with the absolute value of the total inhibitory effect reaching 0.792. Nitrogen regulation had no significant direct effect on growth rate, but significantly affected soil pH and chlorophyll a content. In addition, soil pH had a significant direct negative effect on soil nitrate nitrogen content (−0.55) and a significant direct positive effect on chlorophyll a content (0.54). SOD activity had a significant direct negative effect on growth rate (−0.44).

## 4. Discussion

In this study, we conducted an interactive experiment of soil acidification and nitrogen regulation. We systematically explored the independent and interactive effects of the two factors on the growth, physiological and biochemical indexes of Chinese fir saplings, and clarified their relative importance on Chinese fir growth. This study provides theoretical support and practical basis for analyzing the internal driving mechanism of Chinese fir plantation decline and formulating precise prevention and control measures.

### 4.1. Interactive Effects of Soil Acidification and Nitrogen Regulation on Soil Physicochemical Properties

The stability of soil physicochemical properties is the basis for the healthy growth of trees. In this study, we found that soil acidification, nitrogen application, and their interaction significantly affected soil pH, available nitrogen content, and the availability of other mineral nutrients. This is consistent with the findings of Blanco et al. (2012) and Pan et al. (2023) [3,6], further confirming that the deterioration of soil physicochemical properties is the core inducement of Chinese fir plantation decline.

Long-term management of Chinese fir plantations accelerates soil acidification through litter decomposition and root exudate release [8]. In this study, high acidification treatment significantly reduced soil pH, and this inhibitory effect was more pronounced under low nitrogen conditions. High nitrogen treatment had no significant regulatory effect on the pH of highly acidified soil. We speculated that the highly acidified soil had exceeded the buffer capacity of nitrogen fertilizer: the surface of soil colloids was saturated with hydrogen ions, and the additional protons released by nitrification of ammonium nitrogen in the fertilizer further aggravated soil acidification, preventing an effective increase in soil pH [13].

Available nitrogen (nitrate nitrogen and ammonium nitrogen) is the core nutrient for Chinese fir growth. Its content change directly reflects the soil nutrient supply capacity. In this study, the high acidification treatment significantly reduced soil nitrate nitrogen content, while ammonium nitrogen content increased with the increase in acidification degree under the no nitrogen and low nitrogen conditions. This is consistent with the research results of Yu et al. (2017) [5]. The possible reason is that soil acidification inhibits the activity of nitrifying bacteria, reduces the conversion of ammonium nitrogen to nitrate nitrogen, and leads to the accumulation of ammonium nitrogen in the soil [22,23]. Nitrate nitrogen is easily leached with precipitation, and the leaching effect is enhanced under acidified conditions, which further reduces its content [24].

Although high nitrogen treatment can significantly increase the basic content of soil available nitrogen, the interaction of high acidification and high nitrogen significantly reduces the available nitrogen content. This indicates that soil acidification will weaken the nutrient supply efficiency of nitrogen fertilizer [25]. This finding explains the phenomenon of “fertilization but insufficient nutrients” in the continuous planting process of Chinese fir plantations. That is, soil acidification caused by long-term continuous planting makes nitrogen application unable to effectively improve soil available nitrogen supply, thereby aggravating the growth decline of trees [26].

In addition, there were significant differences in the effects of soil acidification and nitrogen regulation on available potassium and available phosphorus contents. Under high acidification conditions, nitrogen application significantly increased available potassium content. Available phosphorus content was only inhibited by acidification under the no nitrogen condition, and there was no significant difference under the low nitrogen and high nitrogen conditions. This may be because cations in nitrogen fertilizer (such as ammonium ions) exchange with potassium ions on the surface of soil colloids, promoting the release of potassium ions and increasing available potassium content [12]. The content of available phosphorus is mainly affected by soil pH. Low pH will lead to the combination of phosphorus with aluminum and iron ions to form insoluble phosphate, reducing its availability. However, nitrogen application can alleviate the fixation of phosphorus by regulating the soil nitrogen–phosphorus ratio. Therefore, the inhibitory effect of acidification on available phosphorus is offset under nitrogen application conditions [10].

The above results indicate that the effects of soil acidification and nitrogen regulation on soil nutrients are treatment-specific. Rational regulation of nitrogen application level must be combined with soil acidification degree to achieve efficient utilization of soil nutrients.

### 4.2. Effects and Mechanisms of Soil Acidification and Nitrogen Regulation on the Growth of Chinese Fir Saplings

The change in growth rate directly reflects the growth status of Chinese fir saplings. In this study, we found that soil acidification significantly inhibited the growth of Chinese fir saplings, while the alleviating effect of nitrogen application depended on the degree of soil acidification. It was only effective under low acidification conditions, and had no significant effect under high acidification conditions. This core finding clarifies the interactive regulation law of the two factors on Chinese fir growth, and makes up for the deficiency of previous single-factor studies [19,21].

The main mechanisms of soil acidification inhibiting Chinese fir growth can be summarized into two aspects. First, acidification leads to a decrease in soil pH, damages soil aggregate structure, reduces the availability of mineral nutrients, and increases the activity of toxic metal ions such as aluminum and manganese. These effects inhibit the absorption and transport of nutrients and water by tree roots [10,14]. Second, acidification disrupts the balance of physiological metabolism by affecting the photosynthetic system and antioxidant system of Chinese fir, thereby inhibiting growth [21,27].

Chlorophyll content and chlorophyll fluorescence parameters are core indicators reflecting plant photosynthetic capacity [28]. In this study, the interaction of soil acidification and nitrogen had a significant effect on chlorophyll a content, but had no significant effect on chlorophyll b content and chlorophyll a/b ratio. This indicates that acidification and nitrogen mainly affect the synthesis and function of chlorophyll a in the light reaction system of Chinese fir, rather than the overall proportion of chlorophyll.

High acidification treatment significantly reduced chlorophyll a content, PSII maximum photochemical efficiency (Fv/Fm), and actual photochemical efficiency (ΦPSII). This indicates that acidification causes damage to the PSII of the photosynthetic system of Chinese fir, reduces the light reaction efficiency, and makes it unable to effectively convert light energy into chemical energy [21]. Nitrogen application can alleviate this inhibitory effect, and the alleviating effect of high nitrogen treatment is better than that of low nitrogen treatment. We speculated that nitrogen fertilizer can promote the synthesis of chlorophyll a, increase the photochemical quenching coefficient (qP), reduce the non-photochemical quenching coefficient (NPQ), enhance the light energy capture and conversion efficiency of the photosynthetic system, and reduce the loss of light energy in the form of heat, thereby protecting the photosynthetic system from acidification damage [15]. However, the alleviating effect of nitrogen fertilizer disappeared under high acidification conditions. This may be because high acidification leads to a serious decline in root absorption capacity. Even if nitrogen fertilizer is supplemented, it cannot be effectively transported to leaves for chlorophyll synthesis and photosynthetic system repair, and ultimately cannot alleviate growth inhibition.

The antioxidant enzyme system (SOD, POD, CAT) is an important defense system for plants to cope with oxidative stress. Its activity change is closely related to plant stress resistance [29]. MDA content reflects the degree of membrane lipid peroxidation damage in plants [30].

In this study, high acidification treatment significantly increased SOD and POD activities, suggesting that the saplings experienced potential oxidative stress and activated their antioxidant defense systems to scavenge reactive oxygen species (ROS) [21]. Because ROS content was not directly measured in this study, this inference needs further verification. However, CAT activity showed a trend of first decreasing and then increasing with the increase in acidification degree, and reached the lowest value under low acidification conditions. This may be because the accumulation of ROS in the low acidification environment is moderate, and SOD and POD can already meet the scavenging demand. Under high acidification conditions, a large amount of ROS accumulates, which induces the increase in CAT activity and forms a synergistic defense effect.

The regulatory effect of nitrogen application on antioxidant enzyme activities depends on the degree of acidification. Under low acidification conditions, high nitrogen treatment reduced SOD and POD activities and MDA content. This suggests that appropriate nitrogen application may alleviate oxidative stress caused by acidification and reduce membrane lipid peroxidation damage [31]. Under high acidification conditions, the regulatory effect of nitrogen fertilizer weakened. This may be because high acidification leads to the disorder of plant physiological metabolism and the impairment of antioxidant enzyme system function, making the plants unable to respond effectively to nitrogen fertilizer [21]. In addition, MDA content in the low nitrogen treatment was significantly higher than that in the no nitrogen and high nitrogen treatments. This suggests that low nitrogen may aggravate membrane lipid peroxidation damage under acidification conditions; given that the nonlinear change in MDA content was not synchronized with SOD and POD activities, this interpretation should be treated with caution. This also explains the phenomenon in which “insufficient nitrogen aggravates decline” in the continuous planting process of Chinese fir plantations.

### 4.3. Pathway Analysis and Relative Importance of Soil Acidification and Nitrogen Regulation Affecting the Growth of Chinese Fir Saplings

Correlation analysis and the structural equation model (SEM) further revealed the internal pathways and relative importance of soil acidification and nitrogen regulation affecting the growth of Chinese fir saplings. Correlation analysis showed that soil pH was significantly positively correlated with the growth rate of Chinese fir saplings, and significantly negatively correlated with SOD activity. Available nitrogen content was significantly positively correlated with growth rate. These correlations indicate that soil acidification was associated with reduced soil pH, elevated antioxidant enzyme activities, and inhibited available nitrogen supply, all of which co-varied with the inhibition of sapling growth; however, correlation alone does not establish causality, and the underlying mechanisms require further experimental verification. Nitrogen fertilizer alleviates the inhibitory effect of acidification and promotes growth by increasing available nitrogen content and regulating soil pH. The SEM fitting results further verified that soil acidification was the dominant limiting factor for sapling growth. It exerted a significant direct negative effect on the growth rate of Chinese fir saplings, and also produced an indirect inhibitory effect by reducing soil pH and increasing SOD activity; the absolute value of its total inhibitory effect reached 0.792. Nitrogen regulation had no significant direct effect on growth rate, and only produced an indirect effect by affecting soil pH and chlorophyll a content. This indicates that soil acidification plays a decisive role in shaping Chinese fir growth performance, while the effect of nitrogen regulation depends on the degree of soil acidification. This finding addresses the previously unresolved question of the relative importance of soil acidification and insufficient available nitrogen for Chinese fir growth [3,20].

Combining SEM path analysis with the response characteristics of each index, we clarified the core pathways of soil acidification and nitrogen regulation affecting the growth of Chinese fir saplings. Pathway 1: soil acidification inhibits soil available nitrogen supply, damages the photosynthetic system, aggravates oxidative stress, and thus inhibits the growth of Chinese fir saplings. Pathway 2: nitrogen application regulates soil pH and available nitrogen content, alleviates photosynthetic system damage and oxidative stress, and promotes the growth of Chinese fir saplings, but it is only effective under low acidification conditions. These pathways clearly reveal the interaction mechanism of the two factors. That is, soil acidification determines the overall trend of Chinese fir growth. As an auxiliary means, nitrogen regulation can only play a role in the environment with mild acidification, and cannot reverse the growth inhibition caused by high acidification. This provides a key theoretical basis for the prevention and control of Chinese fir plantation decline.

This study systematically reveals the interaction mechanism of soil acidification and nitrogen regulation on the growth and physiological metabolism of Chinese fir saplings, and provides accurate technical guidance for the sustainable management of Chinese fir plantations. However, there are still some limitations. First, the experiment was conducted with Chinese fir saplings. The physiological and metabolic characteristics and responses to environmental stress of saplings and adult trees are different. Whether the results can be directly extended to adult Chinese fir plantations still needs further verification. Second, this study only focuses on the interaction between soil acidification and nitrogen regulation, and does not explore the synergistic effect mechanism of the two factors with other soil nutrients (such as phosphorus and potassium) and microbial communities.

Future research should focus on adult Chinese fir plantations under natural forest conditions to verify the applicability of the conclusions of this study. Long-term positioning experiments should be carried out to explore the combined effects of different soil improvement measures and nitrogen regulation, and screen the optimal management mode suitable for the prevention and control of Chinese fir plantation decline in the red soil region of southern China, so as to provide more targeted technical support for the sustainable management of Chinese fir plantations.

## 5. Conclusions

Soil acidification and nitrogen regulation have significant interactive effects on soil physicochemical properties and the growth and physiological metabolism of Chinese fir saplings. Among them, soil acidification is the dominant factor affecting the growth of Chinese fir saplings, and the alleviating effect of nitrogen regulation depends on the degree of soil acidification. High acidification (acid solution pH 3.5) significantly deteriorated soil physicochemical properties, damaged the photosynthetic system, enhanced antioxidant defense responses, and significantly inhibited the growth of Chinese fir saplings. This inhibitory effect cannot be alleviated by nitrogen application. Under low acidification (acid solution pH 4.5) conditions, the high nitrogen treatment significantly promoted the growth of Chinese fir saplings by increasing soil available nitrogen content, alleviating photosynthetic system damage and oxidative stress. Soil acidification mainly produces direct and indirect inhibitory effects by reducing soil pH and increasing SOD activity, while nitrogen fertilizer produces indirect regulatory effects by regulating soil pH and chlorophyll a content. The results of this study reveal the internal mechanism of soil acidification and available nitrogen supply affecting Chinese fir growth, and provide theoretical basis and technical support for the decline control and sustainable management of Chinese fir plantations.

## Data Availability

The data supporting the findings of this study are included in the article. Further inquiries can be directed to the corresponding author.

## Supplementary Materials

The following supporting information can be downloaded at https://www.mdpi.com/article/10.3390/plants15213248/s1. Table S1. Experimental design of the soil acidification and nitrogen application treatments; Table S2. Chlorophyll fluorescence parameters, including maximum PSII photochemical efficiency (Fv/Fm), actual photochemical efficiency (Fv′/Fm′), photochemical quenching coefficient (qP), non-photochemical quenching coefficient (NPQ), and effective photochemical efficiency (ΦPSII), in Chinese fir leaves under different soil acidification and nitrogen addition treatments.
