# Supplementary material for: The Growth and Physiological Metabolic Responses of Cunninghamia lanceolata Saplings to Interactive Soil Acidification and Nitrogen Application and the Dominant Limiting Factor Determination
Source: Plants (Basel). 2026 Oct 22;15(21):3248. doi: 10.3390/plants15213248 (PMC13765331; doi:10.3390/plants15213248)
Supplement: Supplementary file 1 [file plants-15-03248-s001.zip › plants-4608129-supplementary.pdf]

Supplementary Materials

Table S1. Experimental design of the soil acidification and nitrogen application treatments.

Treatment group	Acidification gradient (acid solution pH)	Nitrogen rate (mg N·kg^{-1} dry soil)	Spray solution
S0N0	Control (distilled water)	0	Distilled water
LSN0	Low (4.5)	0	Acid solution (pH 4.5)
HSN0	High (3.5)	0	Acid solution (pH 3.5)
S0LN	Control (distilled water)	50	Distilled water + LN fertilizer
LSLN	Low (4.5)	50	Acid solution (pH 4.5) + LN fertilizer
HSLN	High (3.5)	50	Acid solution (pH 3.5) + LN fertilizer
S0HN	Control (distilled water)	200	Distilled water + HN fertilizer
LSHN	Low (4.5)	200	Acid solution (pH 4.5) + HN fertilizer
HSHN	High (3.5)	200	Acid solution (pH 3.5) + HN fertilizer

Note: The acid solution was prepared by diluting a mixed acid stock solution of 0.5 mol·L−1 H2SO4 and HNO3 (molar ratio 5:1, v/v) to the target pH. LN and HN indicate nitrogen application rates of 50 and 200 mg N·kg−1 dry soil, respectively; the nitrogen fertilizer was urea (analytical reagent grade, N content ≥ 46.4%). S0, no soil acidification; LS, low soil acidification; HS, high soil acidification; N0, no nitrogen addition; LN, low nitrogen addition; HN, high nitrogen addition.

Table S2. Chlorophyll fluorescence parameters of maximum PSII photochemical efficiency (Fv/Fm), actual photochemical efficiency (Fv′/Fm′), photochemical quenching coefficient (qP), non-photochemical quenching coefficient (NPQ) and effective photochemical efficiency (ΦPSII) in Chinese fir leaves under different soil acidification and nitrogen addition treatments

	Fv/Fm	Fv'/Fm'	qP	NPQ	ΦPS II
S0N0	0.380±0.062	0.346±0.063	0.759±0.120	0.192±0.030	0.256±0.003
LSN0	0.317±0.064	0.299±0.075	0.811±0.125	0.243±0.154	0.247±0.004
HSN0	0.259±0.048	0.232±0.037	0.930±0.041	0.194±0.014	0.253±0.004
S0LN	0.401±0.123	0.378±0.130	0.682±0.234	0.177±0.082	0.241±0.002
LSLN	0.297±0.055	0.275±0.059	0.880±0.078	0.177±0.043	0.256±0.002
HSLN	0.274±0.036	0.259±0.039	0.877±0.056	0.149±0.010	0.247±0.003
S0HN	0.237±0.052	0.228±0.050	0.922±0.048	0.159±0.109	0.248±0.003
LSHN	0.271±0.051	0.260±0.052	0.872±0.082	0.112±0.017	0.2555±0.00
HSHN	0.252±0.040	0.235±0.037	0.924±0.039	0.139±0.020	0.254±0.003
S	0.052	0.851	0.075	0.087	0.225
N	0.054	0.115	0.123	0.187	0.225
S×N	0.232	0.821	0.382	0.312	0.008**

Note: The experimental treatments are: S0, no soil acidification; LS, low soil acidification, HS, high soil acidification. N0, no nitrogen addition; LN, low nitrogen addition; HN, high nitrogen addition; S, soil acidification; N, nitrogen addition; Two-way ANOVA was applied to indicate significant differences among variances. ** indicates significant difference at $p<0.01$.
